# Synergistic CO_2_ Cryotherapy and EGF Delivery for Accelerated Wound Healing Through Anti-Inflammatory and Regenerative Pathways

**DOI:** 10.3390/ijms26188796

**Published:** 2025-09-10

**Authors:** Yongxun Jin, Yong-Hyun Lee, Do Hwan Kim, Caijun Jin, Xinrui Zhang, Jae Ryeong Yoo, Gun-Ho Kim, Dae Hyun Kim, Taek-In Oh, Yi-Sook Jung, Pham Ngoc Chien, Chan Yeong Heo

**Affiliations:** 1 Department of Plastic and Reconstructive Surgery, College of Medicine, Seoul National University, Seoul 03080, Republic of Korea; jinyongxun789@snu.ac.kr (Y.J.); jcjking96@snu.ac.kr (C.J.); zhangxinrui@snu.ac.kr (X.Z.); lionheo@snu.ac.kr (C.Y.H.); 2 Department of Plastic and Reconstructive Surgery, Seoul National University Bundang Hospital, Seongnam 13620, Republic of Korea; 3 Department of Pharmacy, Ajou University, Suwon 16499, Republic of Korea; yhlee@koreansrc.com (Y.-H.L.); jryoo@ajou.ac.kr (J.R.Y.); 4 Korean Institute of Nonclinical Study (KINS), Seongnam 13605, Republic of Korea; dhkim@koreansrc.com (D.H.K.); tioh@koreansrc.com (T.-I.O.); 5 Research Institute of Pharmaceutical Sciences and Technology, Ajou University, Suwon 16499, Republic of Korea; 6 RecensMedical, Inc., Hwaseong-si 18468, Republic of Korea; gunho.kim@recensmedical.com (G.-H.K.); dh.kim@recensmedical.com (D.H.K.); 7 Department of Biomedical Engineering, Ulsan National Institute of Science and Technology, Ulsan 44919, Republic of Korea; 8 Department of Medical Device Development, College of Medicine, Seoul National University, Seoul 03080, Republic of Korea

**Keywords:** wound healing, CO_2_ cryotherapy, epidermal growth factor, anti-inflammatory therapy, transdermal drug delivery

## Abstract

Wound healing remains a significant clinical challenge worldwide, and effective management strategies are essential for improving outcomes. This study investigates the therapeutic potential of the AcuCool™ system, a novel multifunctional device that combines high-velocity CO_2_ cryotherapy with intradermal delivery of epidermal growth factor (EGF), in promoting wound healing. Using a full-thickness skin wound model in Sprague Dawley rats, we compared the effects of Device+EGF treatment to those of conventional microneedling-based EGF delivery and untreated controls. Macroscopic assessments revealed significantly accelerated wound closure in the Device+EGF group. Histological analysis showed enhanced re-epithelialization, reduced inflammatory cell infiltration, and increased collagen deposition. Molecular evaluations further demonstrated downregulation of pro-inflammatory markers (TNF-α, IL-1β, MCP-1) and upregulation of remodeling-related genes including TGF-β1, Collagen I, and Vimentin. In addition, nitrite assays confirmed reduced local nitric oxide levels, indicating suppression of oxidative stress. The AcuCool™ platform offers precise, non-invasive drug delivery with dual physical and biochemical therapeutic mechanisms, enabling superior control of inflammation and tissue regeneration. These findings suggest that AcuCool™ represents a promising therapeutic strategy for accelerating wound healing in acute models. While further studies are warranted in chronic wound settings, this approach may hold translational potential for future clinical applications.

## 1. Introduction

Wound healing is a highly dynamic and precisely regulated process that progresses through four distinct but overlapping phases: hemostasis, inflammation, proliferation, and remodeling. Chronic non-healing wounds, including diabetic foot ulcers and pressure injuries, are still prevalent. Although post-operative wound dehiscence is not invariably chronic, complicated cases may evolve into chronic non-healing wounds. These conditions often lead to prolonged hospitalization, increased medical costs, and substantial reductions in patients’ quality of life [1,2,3,4,5,6].

Current therapeutic strategies include advanced moist dressings [7], platelet-rich plasma (PRP) [8,9], topical or epidermal growth factor (EGF) [10,11], laser therapy, and photobiomodulation [12,13,14]. In addition, microneedle therapy has been explored in pre-clinical models for enhanced drug delivery in wound healing, but has not yet been validated clinically [15]. More recent innovations encompass negative pressure wound therapy (NPWT) [16] and hyperbaric oxygen therapy [17]. While these interventions demonstrate varying clinical efficacy, most rely on single therapeutic mechanisms that may be insufficient to address the multifaceted pathophysiology of chronic wounds. Many treatments face limitations in modulating deep tissue inflammation, ensuring adequate penetration of bioactive agents, or maintaining patient compliance with complex protocols [18,19].

AcuCool™ represents a novel multifunctional wound treatment platform that synergistically integrates localized cryotherapy, digitally controlled CO_2_ spray, and precise intradermal injection of bioactive agents such as EGF. This compact, ergonomically designed handheld device features a precision nozzle system, integrated CO_2_ delivery mechanism, sterile drug interface, intuitive LCD control panel, and automated injection unit. The high-velocity CO_2_ spray achieves rapid, localized cooling that suppresses pro-inflammatory factor expression, reduces edema, alleviates pain, and improves local microcirculation [20,21,22,23,24,25].

The CO_2_-based cooling technology offers significant advantages over traditional liquid nitrogen cryotherapy by providing mild, controllable temperatures that minimize tissue necrosis risk. This approach effectively inhibits inflammatory mediators such as Tumor Necrosis Factor Alpha (TNF-α), Interleukin-1 beta (IL-1β), and Monocyte Chemoattractant Protein-1 (MCP-1) by disrupting NF-κB signaling pathways, facilitating the transition from inflammatory to reparative healing phases [26]. The cryotherapy-induced vasoconstriction-vasodilation cycle creates enhanced local perfusion and oxygenation [27,28,29] while providing substantial analgesic and anti-edema effects that improve patient comfort and treatment compliance [30,31,32]. Simultaneously, the automated injection system delivers regenerative factors like EGF directly into optimal dermal layers, bypassing the limitations of topical applications that often fail to achieve therapeutic concentrations due to barrier function and enzymatic degradation. This precision delivery ensures growth factors reach target cells—keratinocytes, fibroblasts, and endothelial cells—at concentrations sufficient to trigger robust cellular responses including enhanced migration, proliferation, and collagen synthesis [10,11].

Unlike passive dressings or topical applications, AcuCool™ enables unprecedented control over injection depth, dosage, and cooling intensity [18]. The device’s sophisticated monitoring capabilities provide real-time feedback on treatment parameters, enabling immediate adjustments based on tissue response and ensuring consistent, reproducible treatment delivery. This represents a significant advancement in personalized wound care.

This study proposes an innovative dual-action therapeutic strategy that combines the anti-inflammatory effects of controlled CO_2_ cryotherapy with the regenerative potential of intradermal EGF delivery. This approach addresses the multifactorial nature of wound healing dysfunction by simultaneously targeting inflammatory dysregulation, oxidative stress, and compromised growth factor availability. The theoretical foundation rests on evidence that optimal healing requires both appropriate growth signals and a favorable microenvironment that supports cellular responsiveness to these signals. The study methodology encompasses comprehensive evaluation of both biophysical effects of CO_2_ cryotherapy and biochemical responses to intradermal EGF delivery. Assessments include inflammation markers, oxidative stress indicators, and extracellular matrix remodeling parameters. A traditional microneedle plus EGF treatment group serves as a control to demonstrate the integrated system’s clinical advantages. The ultimate goal extends beyond efficacy demonstration to developing evidence-based protocols for optimal device utilization across various wound types, facilitating clinical translation and integration into standard wound care practices.

## 2. Results

### 2.1. Macroscopic and Microscopic Observation of the Wound Healing Process

**Figure 1:**
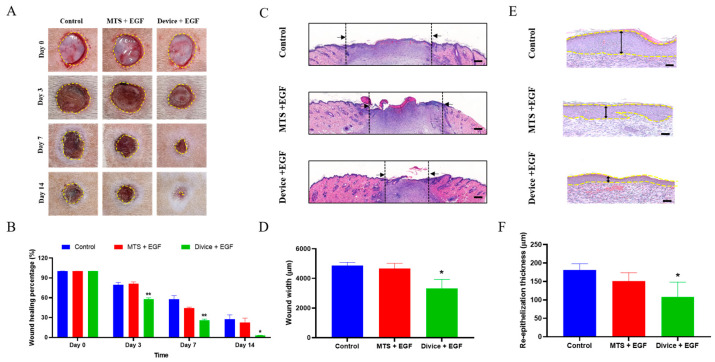
Enhancement of wound healing in rats treated with Device+EGF. (**A**) Representative images of wound healing in the Device+EGF group compared to the control group and the MTS+EGF group. (**B**) Quantitative analysis of wound closure over time. (**C**) Hematoxylin and eosin (H&E) staining of cross-sections of epidermal layers on Day 14; scale bar: 1000 μm. (**D**) Quantitative comparison of wound width on Day 14. (**E**) H&E staining of cross-sections of epidermal layers on Day 14; scale bar: 50 μm. (**F**) Quantitative comparison of re-epidermal thickness gap on Day 14. Representative images are shown; full images are provided in Supplementary Figures S1–S3. The data presented are empirical findings obtained from five rats in each group. Results are shown as the mean ± SEM. Statistical significance was assessed by unpaired two-tailed Student’s *t*-test versus control. * *p* < 0.05, ** *p* < 0.01.

Photographic documentation on Days 0, 3, 7, and 14 revealed progressive wound area reduction across all groups, with both treatment groups demonstrating significantly accelerated healing compared to controls. The Device+EGF group showed markedly smaller wound areas than untreated controls beginning on Day 3 (Figure 1A). By Day 7, both MTS+EGF and Device+EGF groups exhibited significantly reduced wound sizes versus controls, with Device+EGF consistently demonstrating superior wound contraction. This advantage was most pronounced by Day 14 (Figure 1B). Histological analysis using hematoxylin and eosin (H&E) staining on Day 14 tissue sections assessed epidermal regeneration and tissue closure at the microscopic level. Quantitative evaluation revealed that the Device+EGF group achieved significantly narrower wound gaps (3313 ± 279.0 μm) compared to both control (4856 ± 233.3 μm) and MTS+EGF (4665 ± 358.6 μm) groups (*p* < 0.05) (Figure 1C,D). The Device+EGF group’s average wound width was approximately two-thirds that of controls. Furthermore, re-epithelialized epidermal thickness differed significantly among groups. The Device+EGF group demonstrated substantially thinner epithelium (107.5 ± 18.18 μm) compared to the control (180 ± 17.35 μm) and MTS+EGF (150.0 ± 22.89 μm) groups (*p* < 0.05) (Figure 1E,F). These histological findings corroborated the macroscopic observations, confirming the superior healing efficacy of the combined Device and EGF treatment approach.

### 2.2. Effect of the Anti-Inflammation and Antioxidation During the Wound Healing Process

**Figure 2:**
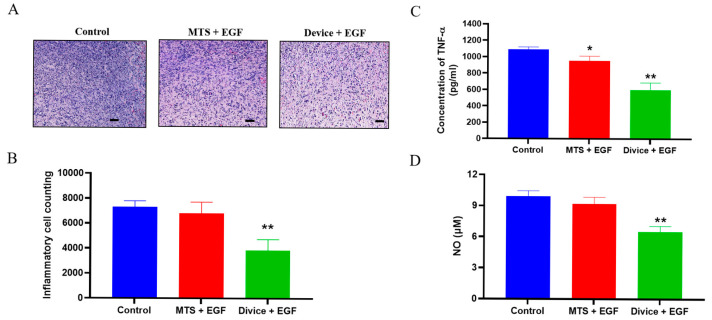
Attenuation of inflammatory response in rats treated with the Device+EGF. (**A**) H&E staining of granulation tissue at the injury site on Day 14 for inflammatory cell counting analysis, scale bar: 50 µm. (**B**) Quantitative results of inflammatory cells on Day 14. (**C**) Nitric oxide (NO) production, quantified using a nitrate assay. (**D**) Concentrations of tumor necrosis factor alpha (TNF-α), determined by ELISA assays. Representative images are shown; full images are provided in Supplementary Figure S4. The data presented are empirical findings obtained from five rats in each group. Results are shown as the mean ± SEM. Statistical significance was assessed by unpaired two-tailed Student’s *t*-test versus control. * *p* < 0.05, ** *p* < 0.01.

Enlarged H&E-stained sections from Day 14 revealed distinct differences in tissue architecture and cellular composition among treatment groups. The control group exhibited minimal granulation tissue formation accompanied by pronounced inflammatory cell infiltration. Both MTS+EGF and Device+EGF groups displayed enhanced fibroblast proliferation with notably reduced inflammatory cell presence, though the Device+EGF group demonstrated the most substantial reduction in inflammatory infiltration (Figure 2A,B).

Biochemical analysis of tissue lysates for pro-inflammatory cytokines and oxidative stress markers provided quantitative confirmation of these observations. The Device+EGF group showed significant reductions in both tumor necrosis factor-alpha (TNF-α) expression and nitric oxide (NO) levels compared to controls (*p* < 0.01). The MTS+EGF group also demonstrated a significant reduction in TNF-α levels (*p* < 0.05) (Figure 2C), but did not show statistically significant changes in NO levels versus controls (Figure 2D).

**Figure 3:**
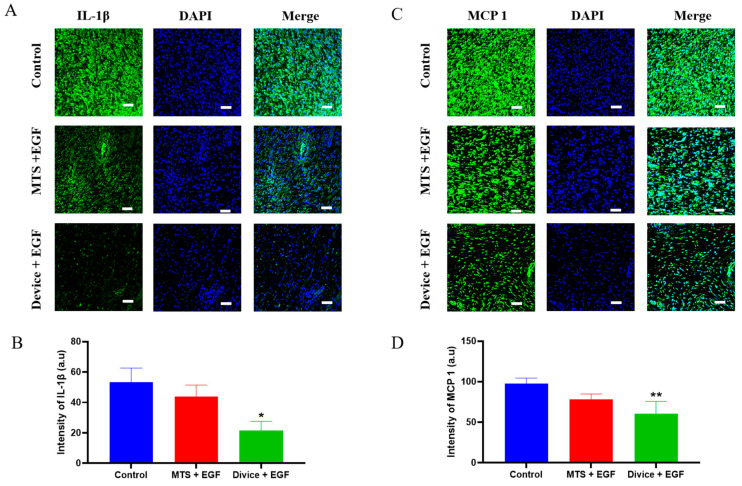
Observations of inflammatory markers in rat wounds using confocal microscopy. Representative immunofluorescence images and quantitative analysis demonstrate: (**A**,**B**) IL-1β expression and quantification, scale bar: 50 µm, (**C**,**D**) MCP-1 expression and quantification scale bar: 50 µm. Representative images are shown; full images are provided in Supplementary Figures S5 and S6. The data presented are empirical findings obtained from five rats in each group. Results are shown as the mean ± SEM. Statistical significance was assessed by unpaired two-tailed Student’s *t*-test versus control. * *p* < 0.05, ** *p* < 0.01.

Immunofluorescence staining further characterized the anti-inflammatory effects during the inflammatory phase by detecting specific cytokine markers. The Device+EGF group exhibited significantly lower IL-1β expression intensity compared to both control and MTS+EGF groups (*p* < 0.05). Similarly, MCP-1 expression was significantly reduced in the Device+EGF group relative to other treatment groups (*p* < 0.01) (Figure 3). These molecular findings corroborate the histological observations, demonstrating that the combined Device and EGF treatment effectively modulates the inflammatory response while promoting constructive tissue repair processes.

### 2.3. Acceleration of the Proliferation and Remodeling During the Wound Healing Process

**Figure 4:**
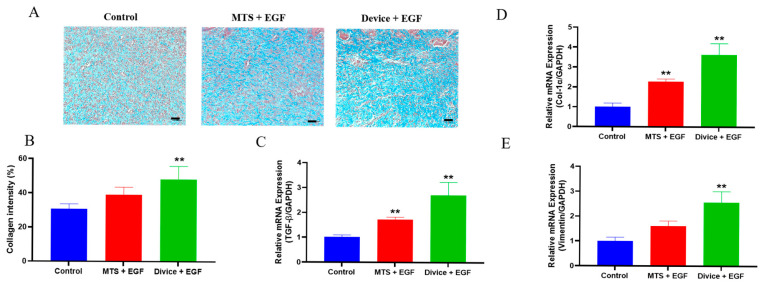
Enhanced proliferation in rats treated with Device+EGF. (**A**) MT staining of granulation tissue on from the injury site on Day 14, scale bar: 50 µm. (**B**) Quantitative results of collagen density on Day 7 and Day 14. Relative expression levels of different cytokines analyzed by RT-PCR: (**C**) TGF-β, (**D**) Collagen I, (**E**) Vimentin. Representative images are shown; full images are provided in Supplementary Figure S7. Results are shown as the mean ± SEM. Statistical significance was assessed by unpaired two-tailed Student’s *t*-test versus control. ** *p* < 0.01.

Collagen deposition assessment using Masson’s trichrome staining revealed substantial differences in extracellular matrix formation among treatment groups. The Device+EGF group exhibited markedly enhanced collagen accumulation, characterized by dense and uniformly distributed blue-stained areas, compared to both control and MTS+EGF groups (Figure 4A). Quantitative analysis confirmed significantly higher collagen content in the Device+EGF group (48.19 ± 3.421%) versus control (30.70 ± 2.915%) and MTS+EGF groups (39.03 ± 4.429%) (*p* < 0.01) (Figure 4B).

To investigate the molecular mechanisms underlying Device and EGF effects during proliferation and remodeling phases, quantitative real-time PCR (qRT-PCR) analysis examined key tissue regeneration genes on Day 14. The Device+EGF group demonstrated significantly upregulated mRNA expression levels of collagen type I, transforming growth factor-beta 1 (TGF-β1), and vimentin compared to both control and MTS+EGF groups (*p* < 0.01) (Figure 4C–E). These findings indicate that the combined treatment enhances extracellular matrix synthesis and tissue remodeling processes at both protein and transcriptional levels, providing molecular evidence for the superior healing outcomes observed in macroscopic and histological analyses.

## 3. Discussion

This study demonstrates through comprehensive macroscopic, histological, and molecular analyses that CO_2_ cryotherapy mediated by the AcuCool™ system combined with EGF delivery significantly accelerates wound healing in rats. Compared to conventional microneedle-assisted drug delivery, this combination strategy exhibits superior efficacy in promoting wound closure, reducing inflammation, enhancing collagen deposition, and facilitating tissue regeneration.

Wound healing represents a complex biological process that progresses through four distinct phases: hemostasis, inflammation, proliferation, and remodeling. During hemostasis, vasoconstriction and platelet aggregation form temporary clots to control bleeding. The inflammatory phase involves neutrophil and macrophage infiltration that clears debris while releasing cytokines and growth factors. The proliferation phase encompasses cellular migration, angiogenesis, and extracellular matrix synthesis to rebuild tissue architecture. Finally, the remodeling phase reorganizes collagen fibers over months to years [3,33,34].

Effective inflammatory phase control is crucial for successful progression to proliferation and remodeling phases [5]. The CO_2_ cryotherapy system achieves beneficial biological effects through precise localized temperature modulation and gas dynamics. Unlike traditional liquid nitrogen-based cryotherapy that may cause nonspecific tissue damage, the CO_2_ cryotherapy system employs controlled high-velocity CO_2_ spray that induces transient vasoconstriction-vasodilation responses. This mechanism enhances local microcirculation and tissue oxygenation, facilitating smooth transition from inflammatory to proliferative phases [26].

The therapeutic mechanisms of CO_2_ therapy extend beyond temperature effects. Through the Bohr effect, CO_2_ reduces hemoglobin’s oxygen affinity, promoting oxygen release to hypoxic tissues [35,36]. Additionally, CO_2_ induces local acidification and activates NO signaling pathways, effectively suppressing pro-inflammatory cytokine expression [21,22]. These combined effects create an optimal microenvironment for accelerated healing while minimizing inflammatory damage to surrounding tissues [27,29].

During the early inflammatory phase, persistent elevation of TNF-α, IL-1β, and MCP-1 is associated with immune dysregulation and chronic inflammation that can impede tissue repair and collagen synthesis [37,38]. This study demonstrated significant downregulation of these inflammatory markers in the Device+EGF group, accompanied by reduced inflammatory cell infiltration. These findings indicate effective anti-inflammatory modulation that improves the wound microenvironment and promotes healing. Furthermore, nitrite assays confirmed that CO_2_ cryotherapy system treatment effectively reduced local NO levels, reflecting decreased inflammatory status and prevention of NO-induced tissue damage.

The Device+EGF group exhibited clear advantages during the remodeling phase. Masson’s trichrome staining revealed markedly increased and uniformly distributed collagen deposition in wound areas, indicating robust extracellular matrix reconstruction capacity. Molecular analysis supported these observations, showing significantly upregulated mRNA expression of key remodeling factors including TGF-β1, Collagen-I, and Vimentin, suggesting enhanced fibroblast activation, migration, and matrix synthesis.

The upregulation of these specific markers carries important biological significance. Vimentin, as a cytoskeletal protein, reflects increased fibroblast activity and cellular remodeling capacity [39,40]. Collagen-I, a core extracellular matrix component, plays crucial roles in improving wound elasticity and mechanical strength [41,42]. TGF-β1 promotes inflammatory and fibroblastic cell recruitment to wound sites while enhancing collagen and fibronectin deposition, ultimately improving tissue architecture and function [43].

The addition of EGF provided synergistic benefits, promoting re-epithelialization as evidenced by epidermal thickening and accelerated wound closure. This demonstrates that the platform delivers therapeutic effects across multiple healing stages by coordinating both physical and biochemical cues. Consistent with existing literature, CO_2_ cryotherapy system has been reported to upregulate VEGF and TGF-β expression, accelerating angiogenesis and epithelial regeneration [33,36,44,45,46]. These findings validate the AcuCool™ system’s potential in promoting comprehensive tissue remodeling and regeneration. Although VEGF expression, microcirculatory dynamics, and oxygenation were not directly assessed, our findings provide indirect mechanistic support for these processes. The downregulation of TNF-α, IL-1β, and MCP-1 indicates effective control of excessive inflammation, thereby establishing a microenvironment favorable for angiogenesis. Concurrently, the upregulation of TGF-β1, Collagen I, and Vimentin suggests enhanced fibroblast activation and extracellular matrix remodeling, processes closely associated with VEGF-driven neovascularization and improved tissue perfusion [26]. In addition, reduced nitric oxide levels imply attenuation of oxidative stress, a key determinant of tissue oxygen balance [47]. Collectively, these results align with previous reports showing that CO_2_ cryotherapy upregulates VEGF, improves microcirculation, and facilitates oxygen delivery, thereby supporting the conclusion that the CO_2_ cryotherapy system+EGF strategy promotes wound healing through synergistic regulation of inflammation, angiogenesis, and oxygenation.

The AcuCool™ system demonstrates significant clinical advantages over traditional CO_2_ therapies, such as water bath immersion and subcutaneous injection [35]. Its non-invasive delivery method eliminates needle-related discomfort and infection risks, improving both treatment safety and patient compliance. The system delivers drugs stably and precisely to the dermis through a streamlined process that enhances reproducibility and pharmacological effectiveness. The high-pressure CO_2_ spray technology provides dual benefits: cryotherapeutic effects and enhanced drug penetration through the skin barrier via powerful physical driving force. This precise control over delivery depth, dosage, and diffusion area reduces inter-individual variability in therapeutic response while minimizing risks of pain, bleeding, and secondary infection [28,30,31,32].

Building on these foundational advantages, the CO_2_ cryotherapy+EGF platform outperforms alternative delivery methods in efficiency, consistency, and clinical convenience. Passive wound dressings depend solely on drug diffusion, resulting in limited penetration, poor dose control, and instability caused by wound environmental factors like pH fluctuations and exudate volume [47,48]. While microneedles partially overcome skin barriers, they remain somewhat invasive and present challenges including micro-injuries at needle sites, uneven drug distribution, and operator dependency [49,50]. Even in a wounded skin model with compromised barriers, microneedle delivery remains limited by operator dependency and uneven distribution. It should also be noted that repeated application of metallic microneedles may induce continuous micro-injury at the wound margins, which could delay healing and partly explain the limited therapeutic benefit observed in the MTS+EGF group. This invasive nature represents a fundamental limitation of metallic microneedles compared to non-invasive platforms such as the CO_2_ cryotherapy system. The CO_2_ cryotherapy system addresses these limitations by using high-speed cooled CO_2_ spray to create non-invasive pathways through temperature gradients and physical pressure. This approach enables rapid dermal drug delivery without mechanical puncture while facilitating the release of bioactive molecules such as growth factors. The synergistic effects of cryostimulation and biochemical signaling provide precise control over delivery depth, velocity, and distribution range, significantly improving therapeutic consistency and stability—making it particularly well-suited for chronic wounds requiring long-term management [26].

While these findings demonstrate the platform’s potential, several limitations warrant consideration. The study utilized an acute wound model in healthy rats, which differs significantly from clinical scenarios involving chronic, non-healing wounds complicated by infection, ischemia, or diabetes. These pathological differences may influence treatment efficacy and limit the direct translation of results to clinical practice. Additionally, the study’s relatively small sample size per group may compromise statistical robustness, and the lack of real-time monitoring for local oxygenation, perfusion dynamics, and VEGF/NO signaling pathways restricts mechanistic understanding of the therapeutic effects observed through histological and molecular evidence. Another limitation is the absence of an EGF-only control group, which would have provided a clearer assessment of the independent contribution of EGF relative to the delivery method.

Future research should address these limitations through several strategic directions. Chronic wound models, particularly diabetic and ischemic wounds, should be prioritized to better reflect clinical conditions. Extended follow-up periods are essential to evaluate scar formation and long-term healing outcomes comprehensively. Advanced imaging techniques should be incorporated to assess vascular remodeling and oxygen supply dynamics in real-time. In addition, including an EGF-only control group in future studies will help to better delineate the specific role of the growth factor from that of the delivery platform. While EGF successfully validated the platform’s delivery capability in this study, expanding research to explore other clinically relevant bioactive molecules—including antimicrobial agents, anti-fibrotic factors, and stem cell-derived exosomes—will be crucial for establishing the system’s broader therapeutic potential and clinical applicability.

## 4. Materials and Methods

### 4.1. System Architecture and Operational Workflow of the AcuCool™ Device

AcuCool™ is an advanced medical device that combines cryogenic cooling based on ultra-low-temperature carbon dioxide with deep transdermal drug delivery functionality. The system primarily consists of a main console, a handheld applicator, a spray nozzle, a drug injection module, and a CO_2_ delivery tube.

The device operates by first regulating ultra-low temperature CO_2_ through a heating mechanism. The conditioned CO_2_ is then delivered via tubing to the spray nozzle, where it is mixed with a therapeutic agent—such as EGF—loaded in a syringe. This mixture is subsequently sprayed at high velocity into the dermal layer of the skin, enabling precise, rapid, and needle-free drug administration.

The operation procedure involves connecting all components of the device, installing the CO_2_ cylinder, and preheating the system. The prepared drug solution is loaded into the syringe and secured onto the nozzle. Once properly fixed, treatment is initiated by pressing the start button. The entire treatment lasts approximately two minutes, during which the system automatically performs the cooling and spraying sequence, and shuts off upon completion.

### 4.2. Animal Experiment

All animal procedures were carefully planned and conducted in strict accordance with the ethical guidelines approved by the Institutional Animal Care and Use Committee (IACUC) of Seoul National University Bundang Hospital (Approval No. BA-2406-393-009-06). The study utilized male Sprague Dawley rats, eight weeks of age, sourced from BioOrient Co. (Seongnam, Republic of Korea), with body weights ranging from 250 to 350 g. Animals were housed in pairs under specific pathogen-free (SPF) conditions, with unrestricted access to standard rodent chow and water. The housing environment was meticulously maintained, with a 12 h light/dark cycle, controlled temperature of 24 °C, and relative humidity set at 55%, ensuring a stable and physiologically appropriate setting for the duration of the experiment.

Adult male Sprague Dawley rats were randomly assigned to three experimental groups (*n* = 5 per group). The first group was treated with a roller-type microneedle system (MTS; Derma Roller System, Shandong Urway Biological Technology, Jinan, China) consisting of 540 stainless steel microneedles arranged on a cylindrical roller, each with a length of 0.25 mm and a sharp conical tip. The device was sterilized with ethylene oxide, disinfected with alcohol, and rolled 4–5 times over the wound surface before topical application of EGF for intradermal delivery. The second group was treated with the AcuCool™ device in conjunction with EGF, and the third group served as an untreated control. Under isoflurane anesthesia, full-thickness excisional wounds measuring 10 mm in diameter were generated on the dorsal skin of each rat using a modified biopsy punch.

Following wound creation, each site was covered with a sterile Tegaderm™ film dressing (3M, Deutschland GmbH Health Care Business, Neuss, Germany) and secured in place using medical adhesive tape. Treatments were administered once daily for the duration of the study. Macroscopic evaluation of the wound healing process was performed on Days 0, 3, 7, and 14, with photographic documentation using a digital camera to monitor visual changes over time. On Day 14, animals were humanely euthanized, and full-thickness dermal tissue samples were harvested from the wound area. The entire circular wound region was excised using sterile 12 mm punch biopsy tools to ensure uniformity and precision in specimen collection for subsequent analysis.

### 4.3. Measurement of the Wound Healing Area

Wound closure percentage was calculated using the equation:

Wound closing area (%) = [(A0 − Ai)/A0] × 100%

where A0 represents the wound area on day 0, and Ai represents the wound area on subsequent days (0, 3, 7, 10, and 14). Wound regions were measured using the ImageJ software program, version 1.54p.

### 4.4. Histological Analysis

Collected tissue samples were fixed in 10% neutral-buffered formalin, followed by a graded ethanol dehydration process (from 80% to 100%), and subsequently embedded in paraffin. Paraffin blocks were sectioned into 5 μm-thick slices for histological analysis. To assess the quality of wound healing at the tissue level, comprehensive histological evaluations were performed. The analysis focused on three primary indicators of wound repair: granulation tissue development, re-epithelialization of the epidermal layer, and collagen deposition, along with the presence and extent of inflammatory cell infiltration.

### 4.5. Hematoxylin and Eosin (H&E) Staining

Paraffin-embedded tissue sections were deparaffinized in xylene for 3 min and rehydrated through a graded ethanol series (100%, 95%, 90%, 80%, and 70%; 3 min each). Sections were then rinsed twice in distilled water for 3 min each. Hematoxylin staining was performed for 5 min to visualize nuclei, followed by water rinses and a brief treatment with bluing reagent for 10–15 s to enhance nuclear contrast. Eosin was applied for 30 s to stain cytoplasmic and extracellular components. Dehydration was completed via sequential immersions in 95% and 100% ethanol for 3 min each, followed by xylene clearing for 1 min, and repeat 3 times. Slides were mounted with coverslips and examined under an Olympus optical microscope for histological assessment of wound healing.

### 4.6. Masson’s Trichrome (MT) Staining

Masson’s trichrome staining was performed using a commercial kit according to the manufacturer’s protocol. Tissue sections were first incubated in Bouin’s solution overnight at room temperature to enhance staining contrast, followed by thorough rinsing with distilled water until tissues appeared clear. Nuclei were stained for 10 min with Weigert’s hematoxylin, followed by rinsing. Sections were then incubated for 10 min in Biebrich scarlet-acid fuchsin to stain cytoplasm and muscle fibers. After rinsing, collagen fibers were differentiated with phosphomolybdic-phosphotungstic acid for 10 min and stained with aniline blue for 10 min. A brief rinse was followed by immersion in 1% acetic acid for 3 min to intensify staining. Dehydration was performed through graded ethanol (100% for 3 min, 95% for 2 min), followed by clearing in xylene for 10 min. Slides were mounted using mounting medium, and collagen distribution was evaluated under a light microscope. Quantification of collagen deposition was performed using ImageJ software (NIH, Bethesda, MD, USA).

### 4.7. Immunofluorescence (If) Staining

Following deparaffinization and rehydration, tissue sections underwent antigen retrieval by microwave heating in 1× Antigen Retrieval Buffer (diluted in 10% FBS/PBS) for four cycles of 5 min each. After cooling, slides were rinsed three times for 3 min each with 1× PBS buffer (pH 7.4) and then blocked for 1 h at room temperature using a blocking solution containing 4% bovine serum albumin (BSA) in 1× PBS to reduce nonspecific binding. Slides were then incubated overnight at 4 °C with primary antibodies targeting IL-1β and MCP-1 (ab315084 and ab7202, diluted 1:500; Abcam, Cambridge, MA, USA). Following primary antibody incubation, sections were washed in PBS and incubated for 1 h at room temperature in the dark with a Goat anti-Rabit IgG Alexa Fluor^®^ 488-conjugated secondary antibody (1:1000 dilution; ab150077, Abcam, Cambridge, MA, USA). After secondary antibody treatment, nuclear staining was performed using DAPI, followed by mounting with antifade mounting medium. Fluorescent images were acquired using a Zeiss LSM 710 or LSM 800 confocal microscope equipped with ZEN software (Zeiss, Oberkochen, Germany), version 3.12 (blue edition). The fluorescence intensity of Collagen I and III was quantitatively analyzed using ImageJ software (NIH, Bethesda, MD, USA).

### 4.8. Protein Preparation

On day 14, harvested tissue samples were mechanically homogenized in a protein extraction buffer containing a protease inhibitor cocktail and 1 mM phenylmethylsulfonyl fluoride (PMSF). Homogenization was carried out on ice for 60 min with intermittent vortexing every 15 min to enhance protein release. The resulting tissue lysates were then subjected to centrifugation at 14,000 rpm for 15 min at 4 °C, allowing for the separation of cellular debris and recovery of the protein-rich supernatant. The supernatant was carefully collected, aliquoted, and stored at −80 °C to preserve protein integrity for downstream biochemical analyses. These included enzyme-linked immunosorbent assays (ELISA) for cytokine quantification, NO measurements.

### 4.9. Enzyme-Linked Immunosorbent Assay (ELISA)

The concentration of rat TNF-α (ab100785, Abcam, Cambridge, MA, USA) was measured by ELISA assay kit following the manufacture guideline.

### 4.10. Nitric Oxide Quantification

Nitric oxide (NO) levels in tissue supernatants were quantified by measuring nitrite, a stable end-product of NO oxidation, using the modified Griess reagent (Sigma-Aldrich, St. Louis, MO, USA). Briefly, 50 μL of each sample was mixed with 50 μL of 1× Griess reagent in a 96-well microplate and incubated at room temperature for 15 min to allow for color development. The Griess reagent was prepared by dissolving 10 g of the modified powder in 250 mL of distilled water and mixing thoroughly by inversion for 5 min, following the manufacturer’s guidelines. A standard curve was generated using 2-fold serial dilutions of sodium nitrite ranging from 0 to 50 μM to enable quantitative analysis. Absorbance was measured at 504 nm using a BioTek Epoch 2 microplate spectrophotometer (BioTek Instruments, Winooski, VT, USA). Nitrite concentrations in experimental samples were calculated by interpolation from the standard curve.

### 4.11. RT-PCR

Total RNA was extracted from harvested skin tissue using RNAiso Plus reagent (Takara Bio, Kusatsu, Japan), in accordance with the manufacturer’s protocol. The purity and concentration of RNA were assessed spectrophotometrically, and 1 μg of total RNA was used for complementary DNA (cDNA) synthesis with the RevertAid First Strand cDNA Synthesis Kit (Thermo Fisher Scientific, Waltham, MA, USA). Quantitative gene expression analysis was performed for Collagen I, TGF-β1, Vimentin, and GAPDH using gene-specific primers. The sequences of the primers were as follows: Collagen I: Forward: 5′-CCG TGA CCT CAA GAT GTG C-3′, Reverse: 5′-GAA CCT TCG CTT CCA TAC TCG-3′; TGF-β1: Forward: 5′-GAC CGC AAC AAC GCA ATC TA-3′, Reverse: 5′-GAC AGC AAT GGG GGT TCT GG-3′, Vimentin: Forward: 5′-AGG TGG ATC AGC TCA CCA ATG ACA-3′, Reverse: 5′-TCA AGG TCA AGA CGT GCC AGA GAA-3′; GAPDH (housekeeping gene): Forward: 5′-TCT CTG CTC CTC CCT GTT CT-3′, Reverse: 5′-ATC CGT TCA CAC CGA CCT TC-3′. Quantitative PCR was carried out using TB Green^®^ Premix Ex Taq™ II (Takara Bio, Kusatsu, Japan) on the QuantStudio™ 3 Real-Time PCR System (Thermo Fisher Scientific, Waltham, MA, USA). The cycling conditions included an initial denaturation at 95 °C for 30 s, followed by 45 amplification cycles of 95 °C for 5 s and 60 °C for 34 s. Relative gene expression levels were calculated using the comparative Ct method (2−ΔΔCt), with GAPDH serving as the internal control.

### 4.12. Statistics Analysis

All quantitative data are expressed as mean ± standard error of the mean (SEM). Statistical analyses were conducted using GraphPad Prism 9 (GraphPad Software Inc., San Diego, CA, USA). Group comparisons were assessed using an unpaired two-tailed Student’s *t*-test. A *p*-value less than 0.05 was considered statistically significant. The levels of significance were indicated as follows: * *p* < 0.05, ** *p* < 0.01 and *** *p* < 0.001. All data collection and analyses were performed under blinded conditions and independently verified to ensure objectivity despite the involvement of device developers.

## 5. Conclusions

This study demonstrates that the AcuCool™ platform, by integrating CO_2_ cryostimulation and epidermal growth factor delivery, establishes a synergistic dual-mechanism therapeutic approach with significant advantages in accelerating wound healing, regulating inflammation, and promoting tissue remodeling in an acute wound rat model. While these findings highlight the potential of this approach, further studies in chronic wound models and clinically relevant conditions are required before its applicability to chronic, non-healing wounds can be established. As an innovative platform that combines physical modulation with biological signaling, AcuCool™ warrants further investigation across broader pathological models and patient populations to evaluate its full translational potential.

## Data Availability

All data generated or analyzed in this study are available from the corresponding author on reasonable request.

## Supplementary Materials

The following supporting information can be downloaded at: https://www.mdpi.com/article/10.3390/ijms26188796/s1.

## Abbreviations

The following abbreviations are used in this manuscript:

EGF	epidermal growth factor
ELISA	enzyme-linked immunosorbent assay
H&E	hematoxylin and eosin
IF	immunofluorescence
IL-1β	interleukin-1 beta
IL-6	interleukin-6
MTS	microneedle therapy system
MCP-1	monocyte chemoattractant protein 1
MT	Masson’s trichrome
NPWT	negative pressure wound therapy
NO	nitric oxide
PRP	platelet-rich plasma
qRT-PCR	quantitative reverse transcription polymerase chain reaction
ROS	reactive oxygen species
SEM	standard error of the mean
TGF-β1	transforming growth factor-beta 1
TNF-α	tumor necrosis factor alpha
VEGF	vascular endothelial growth factor
