# Supplementary figures and images for: Synergistic CO2 Cryotherapy and EGF Delivery for Accelerated Wound Healing Through Anti-Inflammatory and Regenerative Pathways
Source: Int J Mol Sci. 2025 Sep 10;26(18):8796. doi: 10.3390/ijms26188796 (PMC12470236; doi:10.3390/ijms26188796)

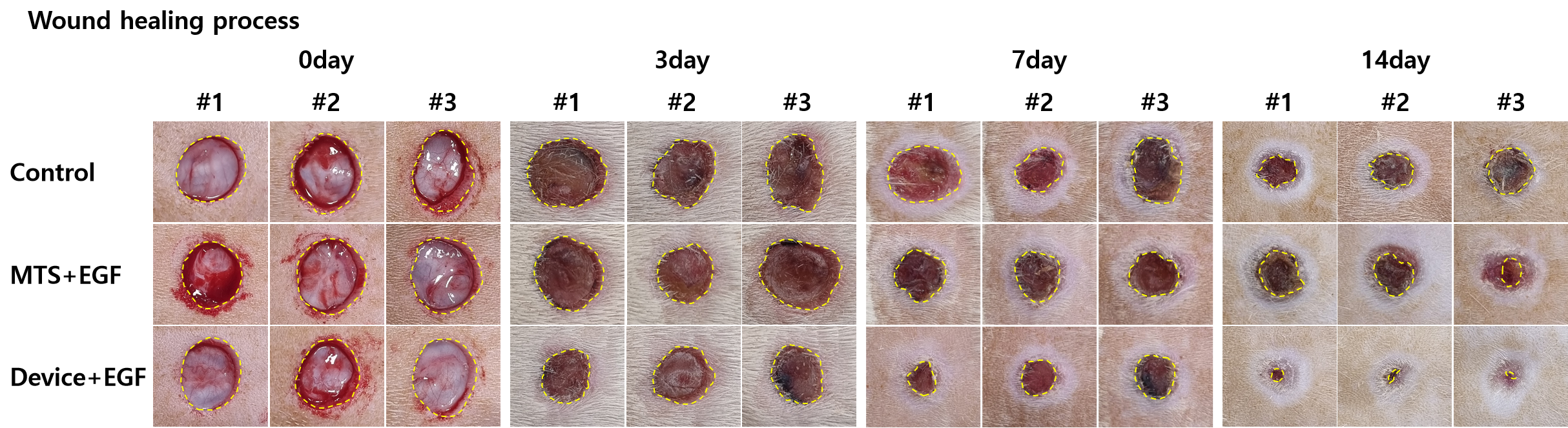

Supplement: Supplementary file 1 [file ijms-26-08796-s001.zip › Supplementary Figure S1(Wound healing process).tif]

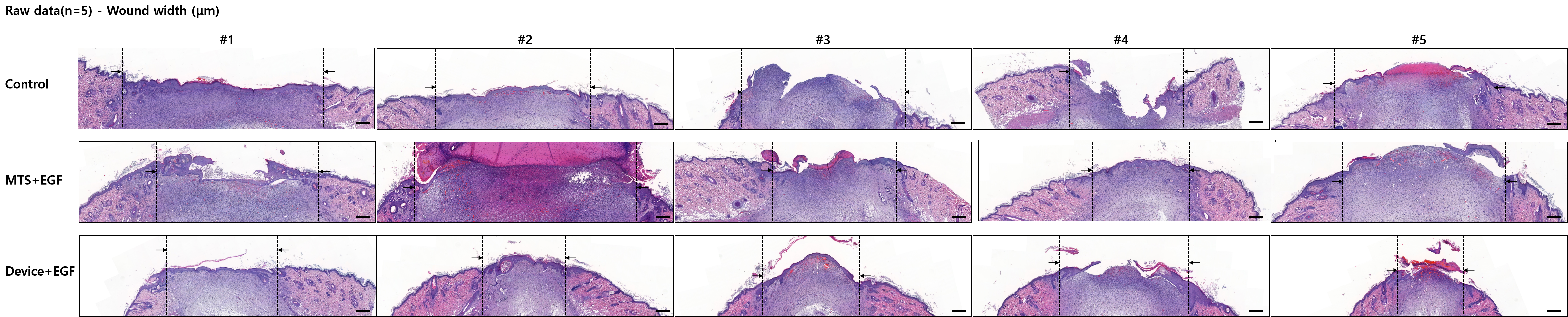

Supplement: Supplementary file 1 [file ijms-26-08796-s001.zip › Supplementary Figure S2(Wound width).tif]

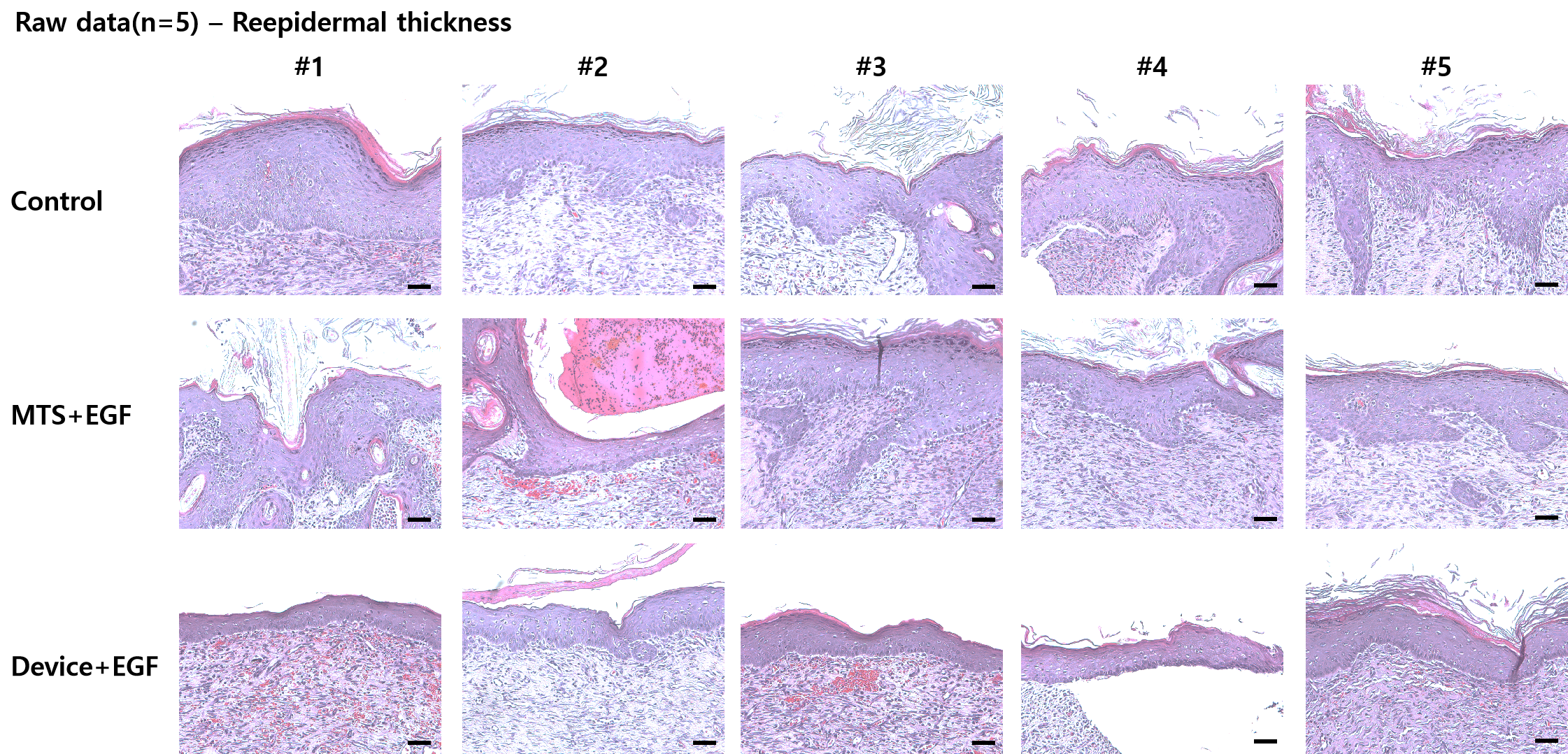

Supplement: Supplementary file 1 [file ijms-26-08796-s001.zip › Supplementary Figure S3(Reepidermal thickness).tif]

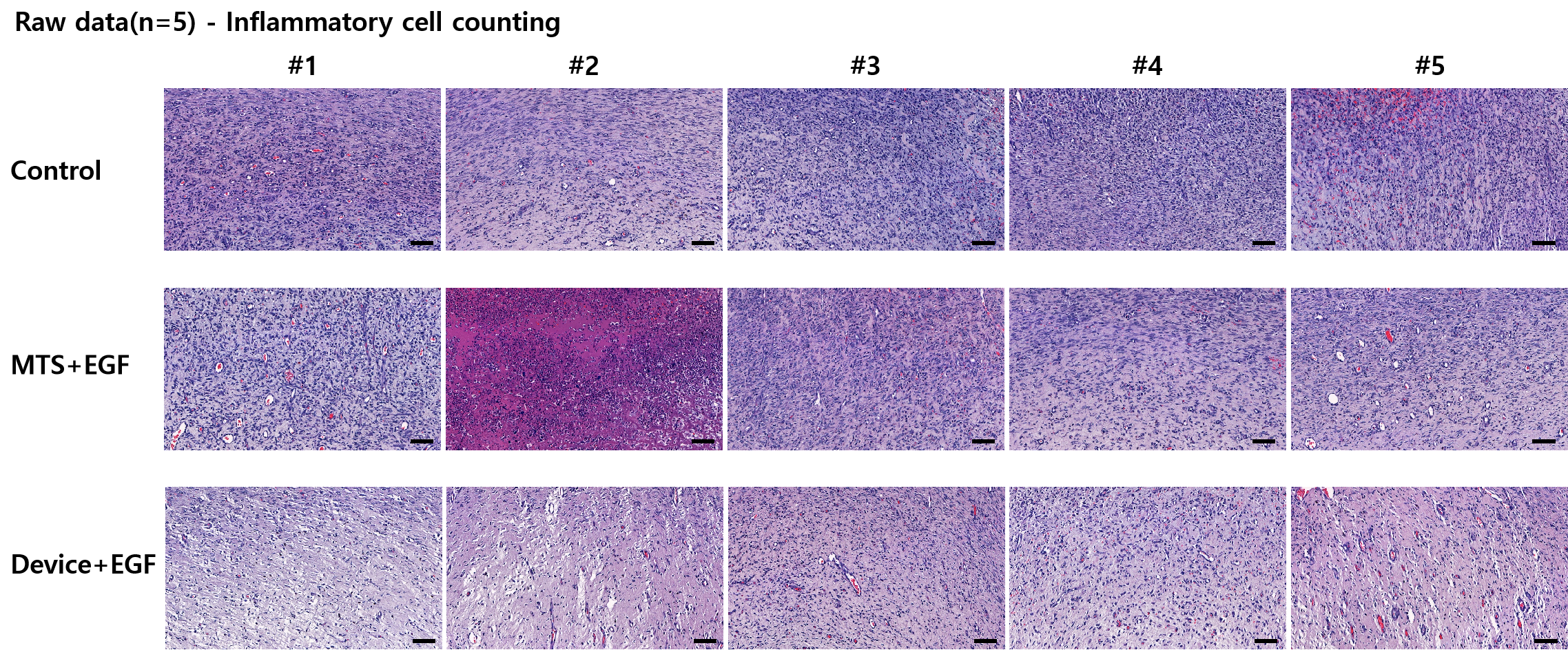

Supplement: Supplementary file 1 [file ijms-26-08796-s001.zip › Supplementary Figure S4(Inflammatory cell counting).tif]

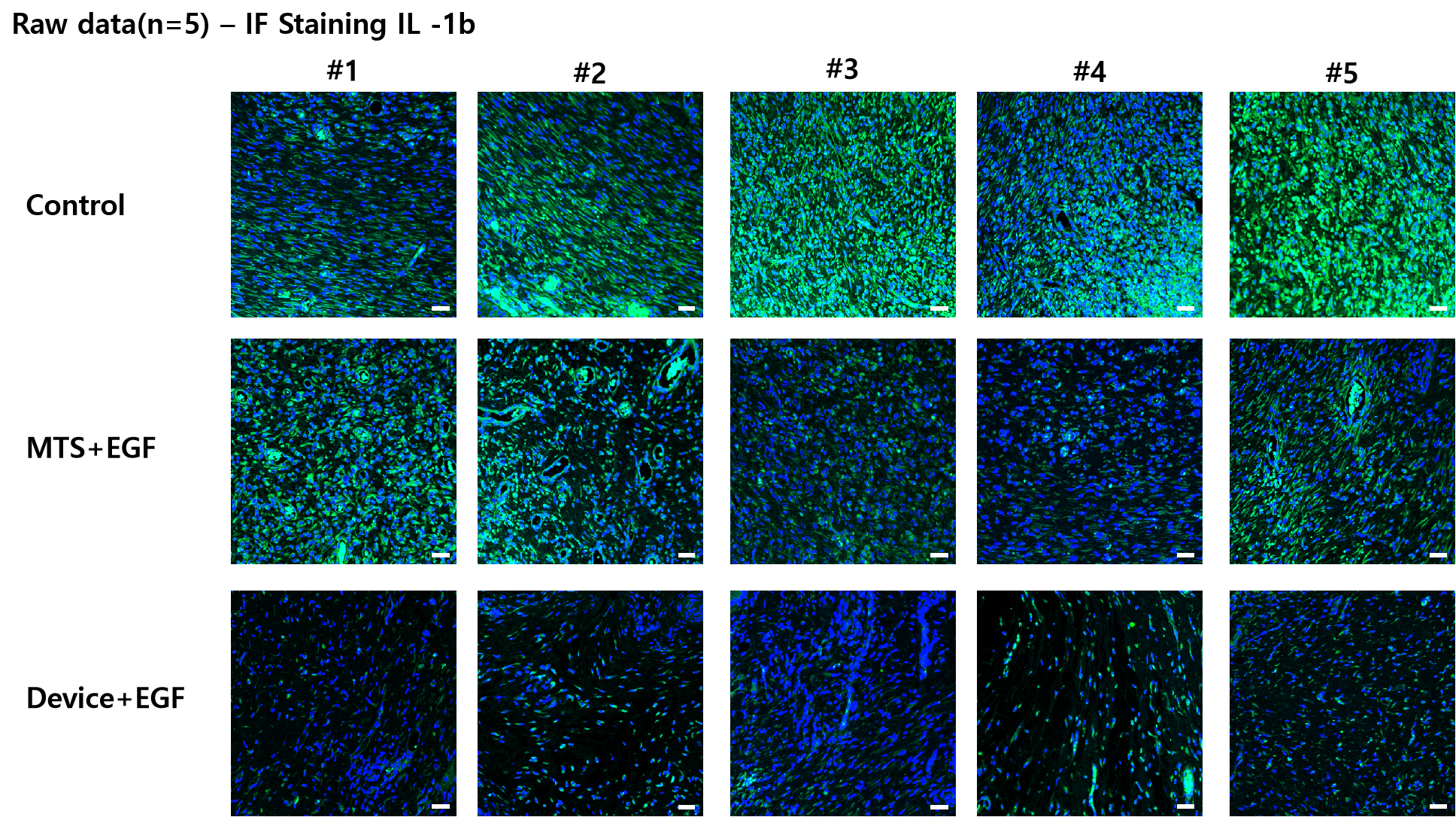

Supplement: Supplementary file 1 [file ijms-26-08796-s001.zip › Supplementary Figure S5(IF Staining IL -1β).tif]

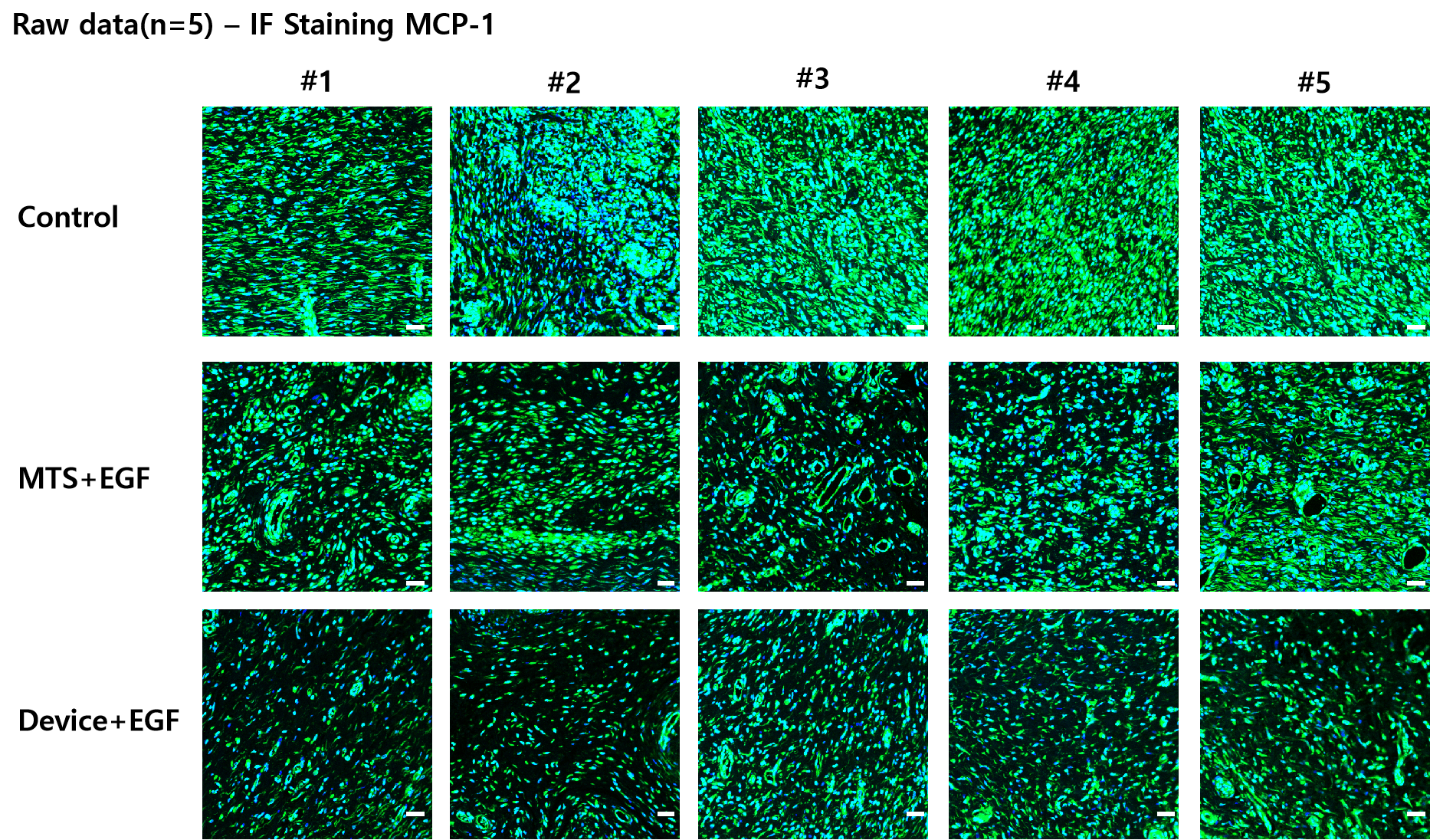

Supplement: Supplementary file 1 [file ijms-26-08796-s001.zip › Supplementary Figure S6(IF Staining MCP-1).tif]

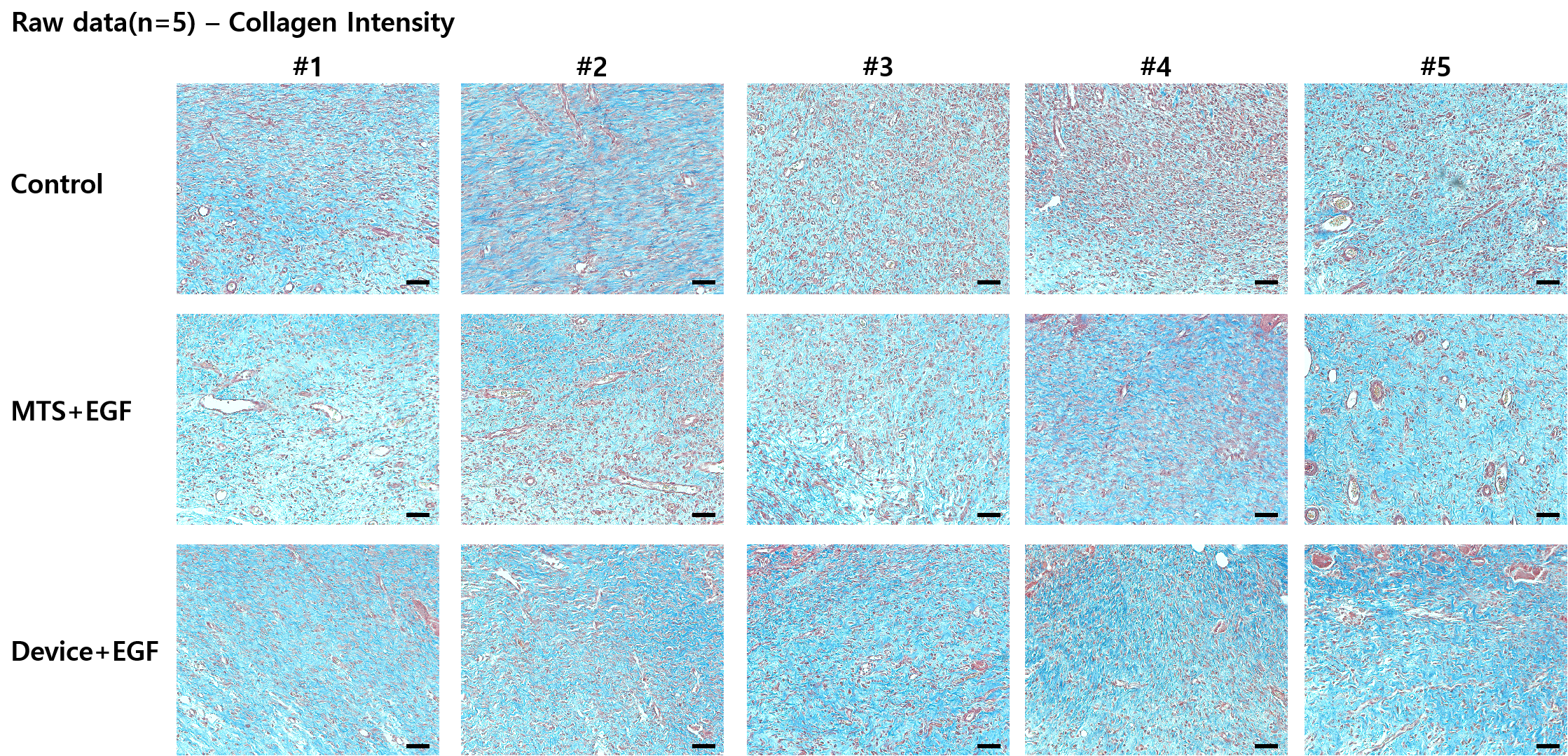

Supplement: Supplementary file 1 [file ijms-26-08796-s001.zip › Supplementary Figure S7(Collagen Intensity).tif]
